# Use of autologous conditioned serum (Orthokine®) for the treatment of the dege-nerative osteoarthritis of the temporomandibular joint. Review of the literature

**DOI:** 10.4317/medoral.18373

**Published:** 2013-03-25

**Authors:** Juan C. Álvarez-Camino, Eduardo Vázquez-Delgado, Cosme Gay-Escoda

**Affiliations:** 1 DDS. Resident of the Oral Surgery and Implantology Master Degree Program. University of Barcelona Dental School; 2 DDS. Associate Professor of Oral Surgery. Chief Professor of the TMJ and Orofacial Pain Unit of the Oral Surgery and Implantology Master Degree Program. University of Barcelona Dental School. Orofacial Pain Specialist of the TMJ and Orofacial Pain Unit of the Teknon Medical Center, Barcelona, Spain; 3 MD, DDS, PhD. Chairman and Professor of Oral and Maxillofacial Surgery. Director of the Master of Oral Surgery and Implantology. School of Dentistry of the University of Barcelona. Coordinator/Researcher of the IDIBELL Institute. Head of the Oral and Maxillofacial Surgery Department and Co-Director of the TMJ and Orofacial Pain Unit of the Teknon Medical Center, Barcelona, Spain

## Abstract

Objectives: Treatment of osteoarthritis (OA) using autologous conditioned serum (ACS) has become in recent years an alternative to consider in the approach of the degenerative joint disease of the knee. There is no support in the literature for the use of ACS for the treatment of OA of the temporomandibular joint (TMJ), although the promising results obtained in human patients with knee joint disease as well as in animal studies are opening the way for its use at the TMJ. The aim of this paper is to conduct a review of the published literature regarding the use of the ACS for the treatment of OA in humans, considering the level of scientific evidence, and following the principles of the evidence-based medicine and dentistry. Material and Methods: A PubMed-MEDLINE search was carried out of articles published between 1980 and 2011. After an initial search, a total of 102 articles were obtained, followed by a selection of the most relevant articles according to the topic; a total of 8 articles were selected, which were stratified according to their level of scientific evidence using SORT criteria (Strength of Recommendation Taxonomy). Results: At the time of this review, there is no available literature referring the use of ACS at the TMJ. However, the use of the ACS in other joints is well documented, both experimentally and clinically, in humans and animals. The reviewed articles, with a level of evidence 1 and 2 according to the SORT criteria, have generally promising results. Discussion and Conclusions: The use of ACS in the treatment of OA in joints other than the TMJ, is endorsed by the level of evidence found in the literature, which opens the door to future studies to determine the feasibility of the use of the ACS in the treatment of degenerative OA that affects TMJ.

** Key words:**Osteoarthritis, temporomandibular joint, autologous conditioned serum.

## Introduction

Osteoarthritis (OA) of the temporomandibular joint (TMJ) is a common pathology. It appears as a degenerative, progressive, slow evolution pathology, which affects TMJ, both hard and soft tissues. OA is associated to mechanical overload of the joint caused by micro or macro trauma, secondary to the presence of other pathological process, such as a disc disease, or associated to a systemic condition of the patient, in the case of rheumatoid arthritis (1). OA is a degenerative disease associated with inflammatory changes of the TMJ. Although associated with all age groups, there is a higher incidence in patients of about 40 years old, with a predilection for women. Radiographic changes of the TMJ associated with OA are present in 17% of patients over 65 years; 50% of those have a mild to moderate degree (or worse) level of pain and dysfunction in their jaw, which would reduce the 17% figure in individuals over 65 years of age to 8.5% with substantial clinical symptoms (1). It is no clear what the prevalence of TMJ OA is in younger population.

The clinical signs of OA include pain, decreased jaw mobility and crepitus in mouth opening (1). OA appears as changes in the surface of the joint, affected by erosion, sclerosis, thinning and the appearance of unilateral osteophytes, although it can be seen affecting both TMJs (2). These changes in the articular surfaces are joined by changes in the synovial fluid, as increased levels of the inflammatory mediators, such as interleukin (IL) 1B, tumor necrosis factor ? and IL 6 (1).

Treatment of symptomatic OA is primarily based on a restrictive therapy of joint movements, the use of drugs such as nonsteroidal antiinflammatory drugs (NSAIDs) and cyclooxygenase-2 (COX-2) inhibitors, the use of occlusal splints, thermotherapy, or minimally invasive techniques such as arthrocentesis. The use of invasive techniques as the arthroplasty is recommended only when all other treatment options are depleted. Pharmacological treatment of OA does not prevent the progression of the disease, although it has been shown to significantly reduce the associated symptoms (3). Additionally, prolonged use of these drugs is associated with significant side effects such as increased risk of gastrointestinal bleeding and cardiovascular ischemia (3). Non-symptomatic OA should be monitored by the specialist, but it doesn’t required symptomatic treatment most of the time.

In recent years, research for the treatment of OA has been focused on the use of drugs that not just improve the patient’s symp-toms, but also be able to alter the development of OA, and therefore delay or even prevent the need for invasive techniques for treatment. Glucosamine sulfate and chondroitin sulfate have been the most studied. It has been shown that, used in combination, have been effective in reducing symptoms in a subgroup of patients with moderate to severe pain in the knee joint (4). Despite this, their role as modifiers of the progression of OA is still controversial.

The balance of the cytokines, along with their receptors and receptor antagonist are crucial, not just for the initiation and progres-sion of OA, but also in the clinical expression of this condition (5); therefore, cytokines have been proposed as targets in the ther-apy of OA. Interleukin 1? (IL-1?), a proinflammatory cytokine, seems to play an important role in the production of collagenase and prostaglandins by releasing a cascade of inflammatory and catabolic events, resulting in a reduction in the synthesis of pro-teoglycans and cartilage-specific collagens (6). The number of receptors for IL-1? is significantly increased in chondrocytes and synovial fibroblasts in OA (7). The natural inhibitor of IL-1?, IL-1? receptor antagonist (IL-1?Ra) may have the potential to limit intraarticular action of IL-1?, and thus, control the development of the disease. In an animal model, stimulation “in vivo” of IL-1?Ra by gene therapy resulted in a significant improvement in clinical parameters of pain and disease development, maintenance of articular cartilage as well as beneficial effects in the synovial membrane and articular cartilage at histological levels(8).

Autologous conditioned serum (ACS) was developed in the mid 90’s, with the idea of obtaining an injectable material enriched with IL-1?Ra, to be used as a treatment for OA (9). Meijer et al. (10) showed that, following the blood exposure to glass beads, a rapid increase in the synthesis of various inflammatory cytokines, including IL-1?Ra, is obtained. ACS is prepared by taking a blood sample and incubating it in a syringe, into which CrSO4-coated glass beads are disposed. It has been shown that the synthesis of IL-1?Ra, as well as other anti-inflammatory cytokines such as IL-4, IL-10 and IL-13 (3) is stimulated through this procedure. Currently, the ACS is marketed under the name of Orthokine ® (Orthogen, Düsseldorf, Germany). ACS theraphy appears as an option in the treatment of non-restrictive OA, in terms of its evolution and associated pain. Nevertheless, surgery should be considered as a treatment option for severe, restrictive OA.

The aim of this paper is to conduct a review of published literature regarding use of the SAC in the treatment of OA in humans, taking into account the level of scientific evidence, and following the principles of based-evidence medicine and dentistry.

## Material and Methods

A PubMed-MEDLINE search of articles published between 1980 and 2011 was conducted. In an initial approach, the search terms “Osteoarthritis,” “TMJ”, “Autologous Conditioned Serum” and “Orthokine” were used, the first of them included in the MeSH (Medical Subject Heading) data base. Search was limited to “in vitro”, animal and human studies, and articles written in English. The terms were then merged in a second search using the Boolean operator “AND”, obtaining the articles that included two or more of the search terms. The authors analyzed the searched articles to verify their relevance with the topic under study. The irrelevant articles were discarded. Next, the authors stratified the items according to their level of scientific evidence, using the SORT criteria (Strength of Recommendation Taxonomy) ( Table 1, Table 2, Table 3). The authors then compared their results, in case of disagreement the results were discussed. Subsequently, according to the level of scientific evidence of the articles reviewed, a recommendation level was declared in favor of, or against the use of ACS in the treatment of OA.

**Table 1:**
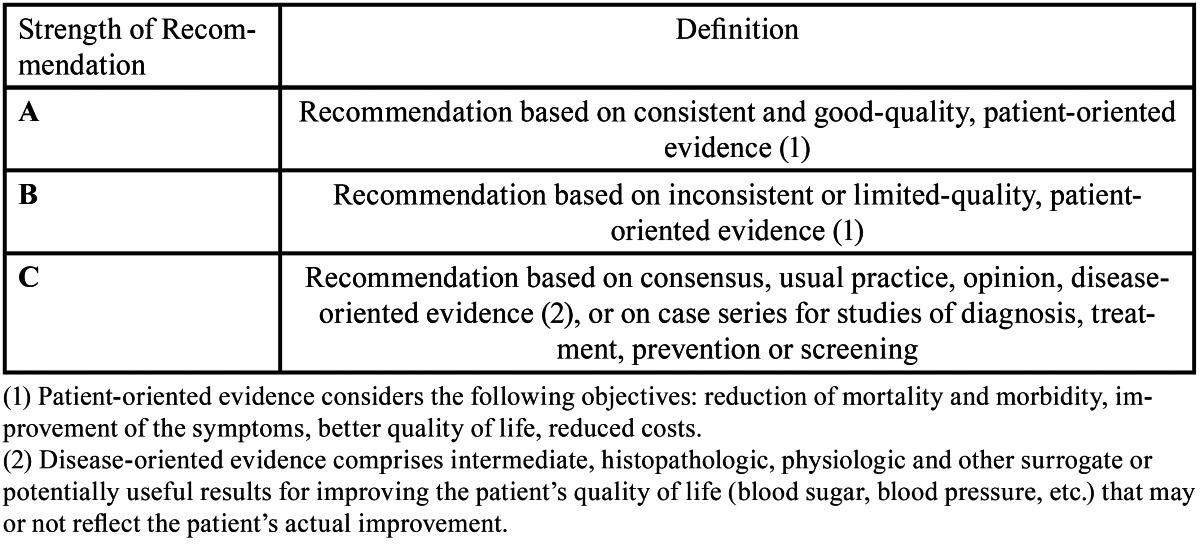
SORT Criteria (Strength of Recommendation Taxonomy).

**Table 2:**
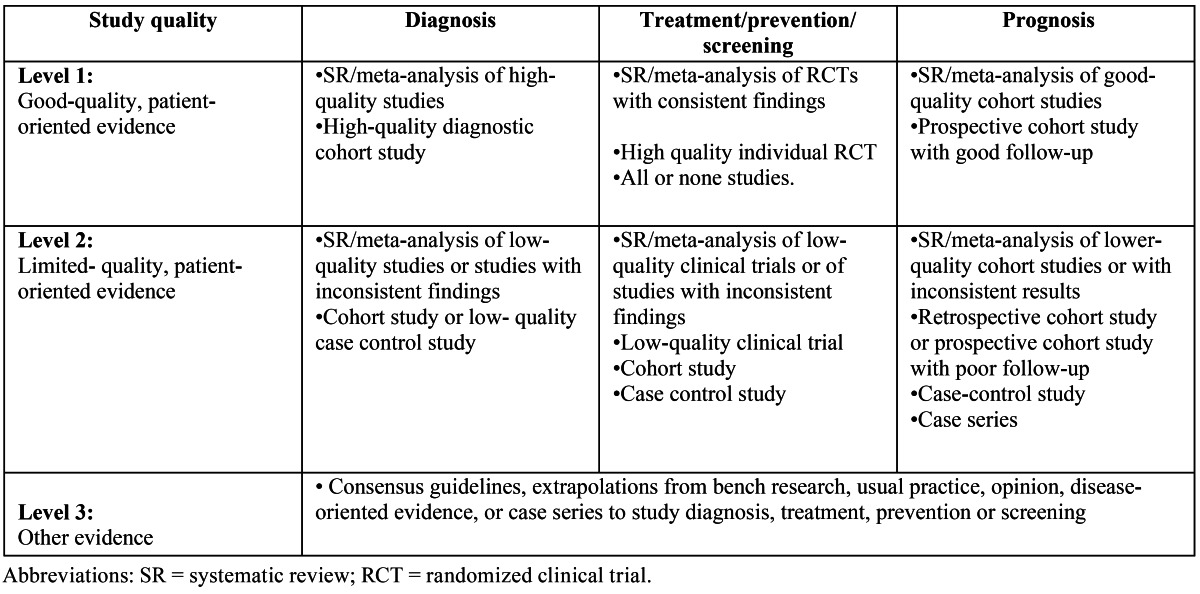
Levels of scientific evidence.

**Table 3:**
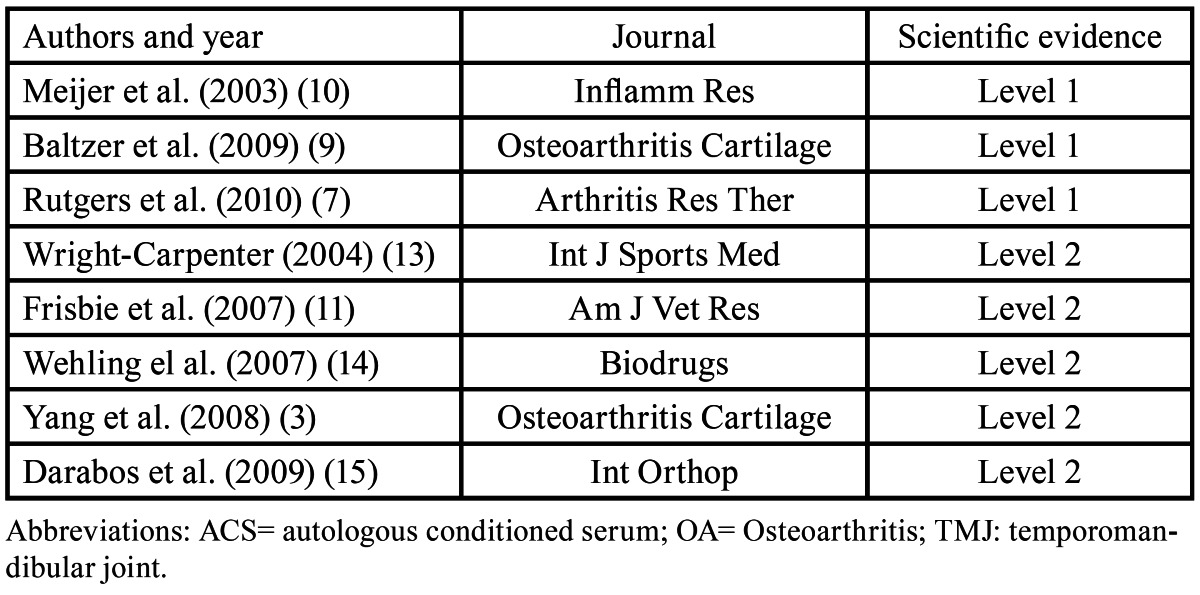
Level 1 and 2 studies that analyze the use of ACS in the treatment of OA of the TMJ.

## Results

The initial PubMed-MEDLINE search provided a total of 43891 articles for the term “Osteoarthritis”, 20440 articles on “TMJ”, 102 for the term “Autologous Conditioned Serum” and 6 items on “Orthokine”. After the second electronic search, which merged keywords, a total of 102 articles with two or more of the terms used were obtained.

Articles with significant methodological errors such as an insufficient patient sample, inadequate sample selection criteria, imprecise definition of the study groups, inadequate description of the analyzed variables, or incomplete and/or inadequate presentation of the results obtained in the study were discarded.

A total of 23 articles with relevance to our review were obtained after the first analysis. These articles were stratified by their level of scientific evidence, using the SORT criteria, discarding those articles with a level of evidence 3. A total of 8 articles were obtained, three articles with a level of evidence 1 and the remaining five with a level of scientific evidence of 2. None of the articles reviewed referred to the use of ACS for the treatment of OA of the TMJ.

In accordance with the principles of evidence-based dentistry, the analysis of the results revealed a type A recommendation for the use of the ACS in the treatment of OA, even with a lack of specific studies evaluating its use at the TMJ.

## Discussion

It is believed that IL-1? plays a role in the cartilage destruction, inherent to articular pathologies such as OA. It has been suggested, both in experimental and animal models of OA, that IL-1?Ra could have beneficial effects associated to the symptoms and structural changes shown in OA. The use of the ACS as a source of IL-1?Ra appears to achieved good results in patients with synovial joints OA, like the knee, although these results may not apply in a straightforward manner to the OA of the TMJ, because to their unique anatomy and physiology. Anyway, these results serve as a reference when determining the efficacy of the ACS in the TMJ treatment.

Baltzer et al. (9) and Yang et al. (3) indicated that use of the ACS is safe and effective in the treatment of OA of the knee joint, and that its therapeutic effects may persist for at least two years. Baltzer et al. (9) explained that, although the mechanism through which it is possible to obtain an increase in the concentration of IL-1?Ra joint level is not entirely clear, ACS is a very reproducible technique, with predictable effects. The clinical effects observed can be explained by a multitude of active therapeutic molecules with synergistic activity, although their persistence over time is not clear.

The therapeutic preparation known by the trade name of Orthokine®, is based on the stimulation of the increased concentrations of inflammatory cytokines, such as IL-1?Ra, inside the joint complex. The technique involves perform of a blood culture of the patient using a syringe that contains CrSO4 surface-treated glass spheres. After a 24 hours incubation at 37 °C, a clearance by centrifugation of the sample is made. The ACS, now enriched with IL-1?Ra, IL-4 and IL-10, is injected inside the affected joint (10).

Exposure of the patient’s blood to the CrSO4 surface-treated glass spheres leads to a vigorous and rapid increase in the concentration of various inflammatory cytokines, including IL-1?Ra, IL-4 and IL-10 (10). An increase up to 140 times the concentration of IL-1?Ra during incubation for 24 hours can be detected (10,11). However, no increased levels of pro-inflammatory cytokines such as IL-1? or tumor necrosis factor ? (TNF-?) were detected in the same period of incubation (10).

The use of ACS therapy has been studied in a profuse way in synovial joints such as the knee. Studies concerning the treatment of OA are especially prolific in horses, because it is considered a high incidence disease among equines, with a great economic importance in this industry.

In a study of Frisbie et al. (11), 16 horses with arthroscopic-induced OA of the medial carpal joint were treated, eight of them (experimental group) by intra-articular injection of ACS previously obtained from a blood sample, and the remaining eight sub-jects (control group) using an intra-articular injection of phosphate buffered saline (PBS), once a week for 5 weeks. The joints treated with ACS showed significant clinical improvement, compared with those in the control group. Moreover, the joints treated with the ACS showed a significant decrease in synovial membrane hyperplasia, as well as a decrease level of intraarticular bleeding, with no statistical significance.

The analyzed literature is limited to the treatment of the OA of the knee joint with the ACS technique in humans. Baltzer et al. (9), in a prospective, randomized, double blind study, of 376 individuals with knee joint OA, reported that, patients treated by ACS showed significant improvements in their quality of life compared to those in who was performed intra-articular injections of hyaluronic acid or placebo. The authors reported that treatment with the ACS is safe, having therapeutic effects over the main clinical parameters associated with OA of the knee joint. The authors added that treatment is effective in OA patients with low or medium pain level, measured by visual analogue scale (VAS); the authors also reported positive results in patients with severe pain, although they recommend not extrapolate the results to all OA patients.

Yang et al. (3), in a multicenter, prospective, double-blind study, evaluated 167 randomized patients who received 6 intra-articular injections, ACS for patients in the experimental group or saline solution for those included in the control group. After carrying out controls at 3, 6, 9 and 12 months, statistically significant improvements in both groups with respect to the values obtained at baseline were observed. Authors showed that symptoms at 3, 6, 9 and 12 months reported by patients treated with the ACS would have better clinical outcomes than those treated with placebo, but clarify that the statistical difference is small. The authors reported the occurrence of complications associated with intra-articular injection of the ACS, as in a case of septic arthritis caused by the injection, and another case in which a patient suffered repeated inflammatory reactions after 3 injections, after which was withdrawn from the study.

The main problem encountered when the transpose of these results is performed into the daily practice of the treatment of OA of the TMJ, is the lack of specific research in our area. All the analyzed studies are the result of the work of scientific teams involved in the treatment of OA of large synovial joints, such as the knee. The physiology and anatomy of the TMJ, a bilateral bicondylar synovial joint, with significant differences in other body joints, so that although the results obtained in other joints are easily extrapolated to the TMJ, these are not specific, and variations may exist in the clinical and statistical results. Zhang et al (12), make a histological evaluation in 24 New Zealand rabbits, performing a partial perforation of the articular disc, thus inducing OA at both TMJs. The intra articular injection of 50 g of human IL-1?Ra solution with 50 ?l of saline into the right TMJ (experimental group) and 50 ?l of saline into the left TMJ (control group) was performed. Animals were sacrificed at 12 and 24 weeks after injection, histological evaluation of the mandibular condyles was performed, followed by a genetic chain reaction (PCR) study. Histologically, fibrocartilage articular thickening in the experimental side and a considerable increase in the number of chondrocytes in comparison with those found in the control side was observed at both 12 and at 24 weeks; Control side showed a considerable increase in the histological changes associated with degenerative OA after 24 weeks. No significant differences in the ex-pression of TNF-? between both groups was observed, although the expression of collagen type II was greater in the experimental side, so they conclude that the activity spectrum of TNF- ? and IL-1 in OA may overlap, the application of recombinant IL-1?Ra does not suppress the expression of TNF-?.

Under normal conditions, the balance between different anabolic and catabolic factors maintains articular cartilage homeostasis. However, in OA this balance tilts in favor of the destruction of the cartilage matrix. It has been shown that IL-1 stimulates the secretion of catabolic enzymes, while suppressing the synthesis of matrix proteins, including collagen type II. Therefore, it would be essential in the process of repair of articular cartilage overexpression the suppression of catabolic factors, the stimulation of secretion of anabolic factors. For this reason, the use of IL-1?Ra obtained by the ACS therapy is presented as a tangible alternative in OA treatment.

## Conclusion

Studies to evaluate a possible application of the ACS technique in the treatment of OA of the TMJ are required. The use of ACS in the treatment of OA is supported by the level of evidence found in the literature in this review, may opens the door to future studies to determine the feasibility of its use in the treatment of degenerative OA affecting the TMJ.
